# Transgenerational transmission of trauma and resilience: a qualitative study with Brazilian offspring of Holocaust survivors

**DOI:** 10.1186/1471-244X-12-134

**Published:** 2012-09-03

**Authors:** Luciana Lorens Braga, Marcelo Feijó Mello, José Paulo Fiks

**Affiliations:** 1 Department of Psychiatry, São Paulo School of Medicine, Federal University of São Paulo, Rua Botucatu 431, 04023-061, São Paulo, SP, Brazil

**Keywords:** Transgenerational, Transmission, Trauma, Resilience, Offspring, Holocaust, PTSD

## Abstract

**Background:**

Over the past five decades, clinicians and researchers have debated the impact of the Holocaust on the children of its survivors. The transgenerational transmission of trauma has been explored in more than 500 articles, which have failed to reach reliable conclusions that could be generalized. The psychiatric literature shows mixed findings regarding this subject: many clinical studies reported psychopathological findings related to transgenerational transmission of trauma and some empirical research has found no evidence of this phenomenon in offspring of Holocaust survivors.

**Method:**

This qualitative study aims to detect how the second generation perceives transgenerational transmission of their parents’ experiences in the Holocaust. In-depth individual interviews were conducted with fifteen offspring of Holocaust survivors and sought to analyze experiences, meanings and subjective processes of the participants. A Grounded Theory approach was employed, and constant comparative method was used for analysis of textual data.

**Results:**

The development of conceptual categories led to the emergence of distinct patterns of communication from parents to their descendants. The qualitative methodology also allowed systematization of the different ways in which offspring can deal with parental trauma, which determine the development of specific mechanisms of traumatic experience or resilience in the second generation.

**Conclusions:**

The conceptual categories constructed by the Grounded Theory approach were used to present a possible model of the transgenerational transmission of trauma, showing that not only traumatic experiences, but also resilience patterns can be transmitted to and developed by the second generation. As in all qualitative studies, these conclusions cannot be generalized, but the findings can be tested in other contexts.

## Background

More than 50 years after the liberation of Nazi concentration camps, researchers and clinicians are still devoted to studying the long-lasting consequences of the traumatic experiences endured by Holocaust survivors and their descendants. This is probably the most comprehensively researched case of transgenerational transmission of trauma [1]. Despite this, there are no published studies conducted with Brazilian offspring of Holocaust Survivors (OHS).

This phenomenon has importance beyond the study of OHS. Many studies suggest that genocides in Rwanda, Nigeria, Cambodia, Armenia, and former Yugoslavia brought about distinct psychopathological symptoms in offspring of survivors [2]. Depression, posttraumatic stress disorder (PTSD), attention deficits, and behavior disorders were more pronounced in children of tortured parents, as compared to controls [3].

In the medical literature, the first study concerning the transgenerational effects of trauma in OHS was published in 1966. The author, Dr. Vivian Rakoff, was researcher at the Jewish General Hospital in Montreal, a city where thousands of Holocaust survivors had settled [4]. Then, other psychiatrists and psychologists who were also treating OHS published case reports of their own [5-7], proposing that the psychiatric disorders of these patients were the result of a “survivor syndrome” [8] perpetuated from one generation to the next [9].

The idea that a parental traumatic experience could reach the second generation soon gained consistency. Clinical studies [10-13] reported a wide range of affective and emotional symptoms transmitted over generations: distrust of the world, impaired parental function, chronic sorrow, inability to communicate feelings, an ever-present fear of danger, pressure for educational achievement, separation anxiety, lack of entitlement, unclear boundaries, and overprotectiveness within a narcissist family system.

Although clinical data provided evidence of psychopathologic effects on OHS, some methodological limitations were apparent: predominance of case reports, unclear definitions of psychopathology, small sample sizes, sampling biases, absence of control groups, and lack of standardized instruments [14].

The literature on the “Second Generation” has grown quickly and profusely since the mid-1980s. Controlled studies have confirmed that Holocaust trauma has psychological impacts on the children of survivors [15], such as higher levels of childhood trauma, increased vulnerability to PTSD and other psychiatric disorders [16,17].

Conversely, other studies have pointed out that OHS were in no way affected in terms of personal adjustment [18-20] and that differences between OHS and control groups could suggest a specific character organization rather than psychopathology [21,22]. Specific types of interpersonal relations were found in OHS, and were related to the pattern of parental communication regarding the Holocaust [23,24]. No evidence of personality disturbances was showed in methodologically sophisticated studies conducted with nonclinical samples of OHS [25,26].

A series of meta-analytical studies conducted with second generation [27] and third generation [28] offspring found no evidence of transgenerational transmission of trauma, except in studies conducted with “selected” samples. These resilient patterns were widely described in literature and, for the purpose of this study, we adopted the definition of resilience provided by the American Psychological Association: “the process of adapting well in the face of adversity, trauma, tragedy, threats, or even significant sources of stress – such as family and relationship problems, serious health problems, or workplace and financial stressors. It means ‘bouncing back’ from difficult experience” [29].

There are three critical conditions in almost all definitions of resilience: “(i) growing up in distressing life conditions and demanding societal conditions that are considered significant threats or severe adversities, (ii) the availability of protective factors, including internal assets and external resources that may be associated with counteracting the effects of risk factors, and (iii) the achievement of positive adaptation despite experiences of significant adversity” [30].

The review of the literature suggests that current studies on transgenerational transmission of trauma to OHS are not conclusive. There is no consensus between the clinical observations and empiric research on the existence of long-term psychological effects on Holocaust survivors and their offspring [31,32]. Whereas case reports are indicative of transgenerational transmission of trauma [33], systematic studies have found no psychopathologic manifestations in the children of Holocaust survivors, except when they were exposed to life-threatening situations [34,35].

The objective of qualitative studies [36-38] is to explore conceptual aspects [39] and understand different meanings and nuances of these apparent contradictions between clinical research and controlled methodologies. The present study aims to detect how Brazilian OHS perceive transgenerational transmission of their parents’ experiences in the Holocaust. We should point out that studying specifically this sample is an important data, in as much as the vast majority of the specific literature is based on American, European or Israelite population. The immigration of the first generation to Brazil and the Brazilian culture itself could play a diverse role in the experience of being offspring of Holocaust survivors.

## Method

### Participants

The nonclinical sample of this qualitative study were recruited initially from a Brazilian Jewish Institution, and then, by *snowball sampling.* The participants and the researchers had no former acquaintance. They were identified by their history of being OHS: they were all men and women whose parents had survived the Holocaust. Specifically, the inclusion criterion for our study was based on the following definition of Holocaust survivor: an individual who had lived under Nazi rule or influence and was subject to: 1) forced displacement; 2) restriction in a ghetto; 3) permanence as a refugee or in hiding places; 4) confinement in forced labor camps or extermination camps [40]. We avoided recruiting patients of mental health services, as the research focus was on the general population.

The participants comprised 15 adult OHS (7 males, 8 females). They ranged in the age from 40 to 66 years and the number of years of education ranged from 12 to 22 years and they were well adapted to their professional careers. At the time of the interview, 13 participants had a stable union or civil marriage and two were divorced. Only one didn’t have children and the main number of children was two. They presented good functioning in terms of personal, social and professional lives. Five of them have had history of prior anxiety disorders, but any participants presented psychiatric problems at the time of the interview. Concerning the parents, nine participants were offspring of parents that had survived Nazi concentration camps and six had parents that had been restricted in ghettos or in hiding places during the Holocaust.

### Procedures

All eligible individuals were sent an invitation letter, which was followed by a phone call from the main investigator (first author), who explained the general aims of the research. Prior to interviews, all participants were assured that their anonymity would be maintained. Then, they completed a demographic questionnaire and provided written informed consent by means of a standardized form approved by the University Research Ethics Committee. The lead investigator conducted all interviews at a location chosen by the participant (usually, at their homes).

Participants were invited to speak freely about the traumatic experiences of their parents and how these experiences affected their lives. The in-depth approach was assured by semi-structured open-ended interviews, which prioritized interviewees’ feelings and the meanings they assigned to their memories and experiences. The interviews were conducted in Portuguese, lasted from one to three hours, were digitally recorded and transcribed *verbatim*. The transcripts were compared to the audiotaped interviews to check for accuracy. The sample was closed when *theme saturation* was achieved.

### Data analysis

A qualitative method seeks to understand and interpret the subjective meanings of peoples’ experiences rather than to test hypothesis and generalize findings. The Grounded Theory (GT) methodology [41], chosen as the theoretical basis for analysis and interpretation of data, is a qualitative research method based on a systematic set of procedures used to develop a model derived inductively from the study phenomenon.

The data analysis involved a succession of phases for its realization. The first stage was actually a *Pre-Analysis*, in which the investigators established an initial contact with the texts without prioritizing any specific aspect, so that the content could gain clarity in an overall perspective.

The second stage consisted of analyzing each interview individually (*Within-case analysis*) and confronting analysis of all cases (*Cross-case analysis*). This procedure enabled discovery of similarities, standards, differences and negative cases [42], what provided the means for the realization of the third stage: *Codification of categories.*

The codification process made possible the creation of categories and subcategories, which were all inductively derived from the content of the interviews. “To code, data are broken down, compared, and then placed in a category. Similar data are placed in similar categories, and different data creates new categories” [43]. To be created, a category or subcategory had to be based in the repetition of the theme in two or more interviews. This process can be better understood as following:

a)  First of all, a more opened way of coding data enabled the identification of *Descriptive Categories*, by recognizing general and non-selective thematic axes in the interviews.

b)  Then, the constant questioning of data led to a new classification of these axes during the phase of axial coding, when data were “put back together in new ways” [44], making new connections and facilitating the emergence of few core phenomena and *Conceptual Categories*.

c)  Finally, the selective coding reordered the categories from the perspective of the core phenomena, enabling the establishment of *Theoretical Categories*.

By this systematic procedure, the interpretative work was accomplished through the development of a comprehensive model to understand the phenomenon in question. An example of data analysis is in Table 1.

**Table 1:** Sample of data analysis: construction of categories

**Coded original transcript**	**Descriptive categories**	**Conceptual categories**
Participant11: *“This secret I carried, I found out that it was their secret* (c1)… *between us, there are no relations of love, there are survival relations* (c2)… *It’s this whole strategy for infusing their children with a manual for surviving a collective disaster. To this day, I bear a trace of this Holocaust survival handbook* (c3)… *in a way, I was told, you have to recognize the signs of a disaster before others do* (c4)”	c1: Transmission of family secrets	c2: Parenting style
	c2: Impaired parental function	c1, c3, c4: Transgenerational transmission of trauma
	c3: Transmission of fear and of a terrifying world view	
	c4: Transmission of mistrust and anticipation of disasters	
Participant 5: “*Each day I tell the story of my father, in this play I am performing in the theatre, it seems I understand a little more* (c5)… *To tell this story in an artistic way – using music, dance, the theatre, painting – is also a way of showing the world what happened* (c6)”	c5: Symbolic dimension of art	c5, c6: Pathways of psychical work over by offspring
	c6: Art as a possibility of representing the catastrophe	
Participant 4: “*My father shows me the story of a man who lived through all those things and is here, right now, moving forward…* (c7) *this attitude reaches me in a certain way. I think I grew up with very few fears”* (c8)	c7: Resilient expressions in parents’ lives	c7, c8: Intergenerational transmission of resilience
	c8: Pathways of resilience in offspring	

To amplify inter-rater reliability of data analysis procedure, three investigators performed the thematic content analysis separately. Then, the individual analysis were compared and discussed, examining the diverse categories and the interpretations of findings. The differences were discussed and a mutual consent was pursued, leading to a high level of agreement between the researchers.

## Results

GT-based analysis of the 15 interviews enabled construction of a system of categories related to transgenerational transmission of trauma and resilience between Holocaust survivors and the second generation (Table 2). Development of conceptual categories led to the systematization of patterns of communication between the first and second generations, and ways in which descendants deal with their parents’ traumatic experiences, which entails mechanisms of transgenerational transmission of trauma or resilience in the second generation.

**Table 2:** Final typology of conceptual categories

**Conceptual categories**	**Subcategories**
1. Communication style	1.1 Open, loving, everyday communication
	1.2 Communication through formal records and documents
	1.3 Indirect communication
	1.4 Catastrophic, fragmented communication
	1.5 Secrets, silence and the unsaid
Experience of trauma	2.1 Terrifying world view: attempts to anticipate disaster
	2.2 Psychical deterritorialization: lack of rootedness and sense of belonging
	2.3 Presentification of the traumatic parental
	2.4 experience
	2.5 Experiences of guilt, victimization and submission
	2.6 Fear of being recognized by external identifiers
	2.7 Attempts to explain parental survival and impact on the second generation
Mechanisms of psychical working over and resilience	3.1 Search for a radical singularity from the parental history
	3.2 Visitation of sites related to the traumatic parental experience
	3.3 Art as a possible means of representing the catastrophe
	3.4 Sense of belonging to a group: bonding and social support
	3.5 Defense of universal, humanistic values

### Communication style

The following subcategorization scheme respected the relevance of modes of communication between Holocaust survivors and their offspring. These modes cross a broad spectrum, ranging from open, everyday communication to its opposite – silence, secrets and the unsaid. Communication style has a significant impact on the manner in which members of the second generation integrate their parents’ traumatic experiences into their lives.

#### Open, loving, everyday communication

Some participants claimed to have had contact with their parents’ experiences through everyday conversation. The presence of an open, loving communication style enabled creation of symbolization mechanisms, which, in turn, favored resilient outcomes.

"My mother would tell us stories of the war ever since I was a child. We’d hear those stories at bedtime. She didn’t tell us fairy tales, but parts of her own story, which always had a [message] of hope. […] She managed to stay in this bubble […] sort of like “Life Is Beautiful”. (Interviewee 01)"

The use of linguistic resources, such as jokes and comic tirades, enables a peculiar approach to the traumatic experience: while it makes the issue easier to address, it simultaneously serves to denounce the violence experienced. In these cases, humor may be viewed as a sort of symbolic displacement, at once allowing the survivor to present and repudiate the traumatic experience without distancing himself or herself from it, creating something of a cushion to lessen the impact of traumatic experience:

"We’re even famous for this Jewish brand of humor, which can find the fun even in a major tragedy, but not in a meaningless way, of course. […] We got to know my father’s history during the war through these ironic, comic outbursts [of his]. […] And then the story came. (Interviewee 05)"

#### Communication through formal records and documents

Many survivors took part in formal interviews and other types of documentary records in an attempt to systematize and order their experience, so their children and grandchildren could be aware of their experiences during the war. Use of this resource by survivors attenuates possible hurdles to direct communication. Many OHS were only able to find out about their parents’ traumatic experiences through access to these records.

"She never talked about this matter. When she recorded [her testimony] for Spielberg’s project, she talked two tapes’ worth… and she wouldn’t talk about it before. […] At all. I only found out about [these] things when I heard the tape. (Interviewee 12)"

#### Indirect communication

Some participants reported that their parents had never directly recounted their traumatic experiences. These OHS became aware of their parents’ trauma indirectly, upon hearing conversations between their parents and third parties, perceiving the meaning of nonverbal manifestations and feeling the weight of the psychical environment during encounters between survivors. The following segments deal with this aspect of parental communication.

"When my mother sat down with her friends, she made a point of having me stay and listen […] She practically forced me to stay. […] It seems she got this morbid pleasure out of talking about it […] She liked to talk about the subject, but not to me, not with me. (Interviewee 07)."

#### Catastrophic, fragmented communication

Some descendents were able to identify a presentification of catastrophic events in their parents’ communication patterns, expressed as aggressive or fragmented discourse, conveying a terrifying view of the world.

"We talked of the Nazis at home as we talk of the air we breathe. […] The stories were about the Nazis. There were few stories about what it was like at the village where my father was born, or what it was like in the city. […] All the stories were about atrocities she’d witnessed. […] We didn’t eat soup at home, we had blood instead. (Interviewee 11)"

#### Secrets, silences and the unsaid

The communication patterns of survivors are often pervaded by feelings of guilt and shame or by fear of having a negative impact on the lives of their offspring. This commonly led to silence, secrets and points left unsaid concerning traumatic experiences. As parents did not address their traumatic experiences, the second generation found it more difficult to achieve psychical and biographical integration of these events, as indicated by the following excerpt:

"My parents don’t build any theories and they have nothing to tell, because they don’t want to remember, they’re afraid of remembering, they’re ashamed of remembering, because each and every one of their memories comes wrapped in this shame of having been [who they were] or having lived what they lived. (Interviewee 01)"

### Experiences of trauma

The following subcategories comprise second-generation experiences that correlate with the disruptive parental life event. Experience of trauma for OHS may be presented as a wide range of manifestations, all of which feature the presence of behaviors, worldviews or experiences that follow patterns similar to the traumatization suffered by Holocaust survivors.

#### Terrifying world view: attempts to anticipate disaster

Some participants claimed their parents had failed to provide an affective framework of security, stability and predictability; instead, many survivors transmitted a terrifying view of the world to their children. The offspring of these parents feel the need to always be ready to react to imminent catastrophes and potential threats to their survival. The following excerpts provide prime examples of this scenario:

"You have to be successful, have power, clout, [to] be able to get by in a disaster situation. […] To this day, I bear a trace of this Holocaust survival handbook […]: if I’m at a meeting with human beings, I’ll never be among the last to leave. Why? Because, in a way, I was told, you have to recognize the signs of a disaster before others do. (Interviewee 11)"

"Expired passport: not an option. It doesn’t matter if you’re not going to travel for the next twenty years […]: you have to renew it, you always have to have, let’s say, a pair of underwear and a pair of socks at hand so you can take off. Always ready. (Interviewee 13)"

#### Psychical deterritorialization: lack of rootedness and sense of belonging

This category describes experiences of feeling a lack of rootedness or belonging. Interviewees associated these feelings with a fragmented family history, characterized by a large number of deceased relatives, unmet relatives, lost roots, difficult logical nexuses, and a precarious, frayed symbolic fabric. It was reported by many OHS as an experience of psychical deterritorialization:

"My mother would tell us how she hid underneath the train: on top of the wheels, under the floorboards. That’s really the image I was raised in, not inside of anything, no. Outside. Outside, in this implausible place in which we travel through life, on top of wheels, with no floor. (Interviewee 11)"

#### Presentification of parental traumatic experiences

OHS related feelings of fear and symptoms of autonomic hyperreactivity, as if they themselves were experiencing their parents’ traumatic events or had lived at the time in which these events transpired. Some interviewees felt as if they were currently living experiences typical of war victims:

"I was having a lot of these nightmares about being chased. Then I told my mother: “I’m going to therapy, I must have a problem”. [In the nightmares,] I ran and I ran and I saw [Nazi] uniforms, narrow hallways and doors and people threatening me with guns. (Interviewee 09)"

#### Experiences of guilt, victimization and submission

Some descendents related to the victimization of their own parents and reported feelings of guilt and submission of their own.

"What was always very tough was how to relate to this story, moving through this issue of being a victim, you know? Myself, that is. Me, as a victim of this history. I have a mother who went through the war and, consequently, I’d be entitled to this host of frailties, issues, problems – anyway, the world would have to be understanding, right? […] Once, my therapist yelled at me, in a situation I couldn’t work out: “How long are you going to lick the Nazis’ jackboots?” […] It’s a [feeling of] great helplessness. (Interviewee 01)"

#### Fear of being recognized by external identifiers

Several interviews revealed transgenerational transmission of feelings of persecution, unfounded on concrete bases or motivations of OHS’ current lives. These may occur as fears restricted to self-identification as a Jew or relative to any type of social identifiability, as the following excerpt shows:

"People wear crucifixes, Stars of David, they put bumper stickers on their cars… I notice I don’t like to be externally identifiable as having any sort of affiliation. […] That’s an expression of “not wanting to be seen”. These are typical thoughts of a survivor’s daughter. (Interviewee 02)"

#### Attempts to explain parental survival and impact on the second generation

Descendents mentioned several elements that could have explained or justified the survival of their parents: chance and luck; contact with powerful, influential individuals (social capital); multilingualism (cultural capital); the opportunity of being assigned distinct tasks or provided a different diet while in concentration camps (access to privileges); attempts to never stand out (making oneself mediocre and “invisible”); and several others.

The descendents’ discourse reveals ways in which these signifiers tied to parental survival were passed down in the form of a family myth, reactualized in the descendents’ lives:

"The Germans came back and killed everyone who was trying to get food. As my father was very small and very weak, he just watched. So, he saw everyone be killed and he wasn’t killed himself. So, that may have given me, and perhaps even my brother, something like this: we never expose ourselves, we never put ourselves out there. (Interviewee 14)"

### Mechanisms of psychical working over and resilience

This category comprises mechanisms used by descendents for psychical working over of parental traumatic experiences. It encompasses a broad spectrum of mechanisms, ranging from the private to the universal. On one pole, descendents can support their own resilience by constructing unique narratives of their parents’ history. On the other, personal involvement in the defense of collective, universal values, transcending the particulars of one’s family history, may be identified as a means of escaping the traumatic experience.

#### Search for a radical singularity from the parental history

Faced with the fragmented, enigmatic discourse of their survivor parents, some interviewees created their own narratives of parental experiences as a means of taking possession of this past. This may constitute a possible resilience mechanism. Sometimes, the process began in childhood:

"I knew there was something odd about my father when I saw that number tattooed on his arm. When I was a child, […] I always asked him and my father would say: “It’s from the war!”. […] Romantically, I used to think: “My father fought in the war! He’s a big hero!” (Interviewee 05)"

This kind of imaginary reconstruction (creation of epic, heroic, poetic, romanticized narratives) of parental history had an actual effect on reality, turning a “cursed inheritance” into a memorable, singular history.

"I think he survived because he’s an only child, and I think he was raised with a lot of love. That saved him and protected him. […]. It’s a private experience. […] In this sense, war strengthened life. (Interviewee 04)"

#### Visitation of sites related to the traumatic parental experience

Field visits enabled displacement of the traumatic experience from a distant, enigmatic, cloudy, inappropriable plane to another, more concrete, accessible and real one. This opened the door to resignification and a symbolic working over of the traumatic parental experience through visitation of parents’ birthplaces and of concentration camps.

"I had the opportunity to go back to Poland with my father. […] It was a very intense experience, because we went to the camp where he was held, we paid a visit to Auschwitz-Birkenau. […] This trip was a very strong experience, because he told me the story again and he worked this story over for himself. (Interviewee 02)"

#### Art as a possible means of representing the catastrophe

Some reports mentioned the crucial role of art (theatre, music, fiction, cinema etc.) in symbolically working over traumatic parental experiences.

"I was very shocked by the play. […] The entire second act took place between two survivors who were moving stones around. […] I took my father [to see it]. When he left the theatre, he said: “That’s my story exactly… that was my job at the camp”. I didn’t know yet that. I was fifteen years old. I remember leaving [the theatre] and writing a short story about that moment, which I kept under lock and key for years. (Interviewee 05)"

Faced with unspeakable trauma, fictional recreation and other forms of artistic output and realization were privileged means of resilience reported by the second generation:

"I asked my father for permission to write his story [of life during the Holocaust]. Each day I tell this story in my play, which I have performed for nearly four years, it seems I can understand a little more. (Interviewee 05)"

#### Sense of belonging to a group: bonding and social support

Opposition to any sort of uniqueness of the parental history ensures comfort in one’s identity, social bonds, and the existence of peers with whom OSH can share their experience.

"We have a family ritual my father used to have […]. Once a year, we visit the graves at the local cemetery. So, we pay a visit to our dead relatives and I always stop by the monument to the Holocaust dead. […] We used to do this and now I do it and my son goes with me and he does the same. (Interviewee 02)"

In this sense, Judaism, as a category of belonging to a group, was not reported by participants merely in its religious, ethnic, linguistic or cultural dimension, but as an existential issue or legacy that cannot but be transmitted.

"The effects of this loss [referring to her father’s trauma] reach me through this search for a Jewish identity, which I ask like so: what is it like to be a Jew? I have this legacy I can’t lose, and that doesn’t mean preaching about the Holocaust, it doesn’t mean bonding with other Jews and saying “poor us”, it doesn’t mean having a relationship with Israel, it doesn’t mean being religious. (Interviewee 02)"

#### Defense of universal, humanist values

Descendents who defend values such as freedom, tolerance and respect for differences tend to escape victimization. As they do not constrain themselves to a uniform, narrow-minded cultural environment, these OHS broaden their worldview and begin to take into account the history of other peoples that have also been subjected to traumatic experiences that, from a humanitarian standpoint, are just as relevant as the plight of the Jews.

"It really pisses me off when someone says something discriminatory. Even when a Jew says: “because we, the Jews, the chosen people”… Who aren’t a chosen people? Blacks aren’t? […] When we go out into the street, we see everyone’s Holocaust. [The homeless] are just as imprisoned as if in a [concentration] camp; society looks, but doesn’t see. (Interviewee 05)"

By not identifying themselves as heirs of an unique traumatic history, descendents give themselves more favorable conditions for developing resilience.

"My father never victimized himself. I think he read the war mainly as a political issue. […] He felt that a fairer world and freedom of thought would avoid that sort of madness […] and that human beings are entitled to freedom and equality. (Interviewee 04)"

## Discussion

Analysis of interviews revealed multiple and diverse effects of transgenerational transmission of trauma, a phenomenon that showed to be neither linear nor one-dimensional. This result was in line with a recent finding in literature, which demonstrated a mixed functional profile in the midlife of OHS: specific strengths (a higher sense of well-being) intertwined with specific vulnerabilities (more physical health problems) [45].

Using the GT-derived conceptual categories, we sought to present a scheme linking transgenerational transmission patterns or resilience patterns to phenomena related to parental trauma. These phenomena were related to three elements: (i) modes of parental psychic *working over*, (ii) ways in which survivors *communicate* the traumatic message to their offspring, and (iii) *repercussions* of trauma on second-generation experiences (Table 3).

**Table 3:** Model for transgenerational transmission: experience of trauma and resilience patterns

**Phenomena associated with parental trauma**	**Patterns of transgenerational transmission**	
	**Experience of trauma**	**Resilience patterns**
1. WORKING OVER (by survivors)	Inability to work over:	Psychical working over ability intact:
	· psychopathological disorders	· personal narratives
	· somatic symptoms	· documentary records
		· cultural rituals
		· collective memory
		· defense of universal values
2. COMMUNICATION (from survivors to their offspring)	· indirect communication	· open, loving, everyday communications
	· fragmented discourse	
	· silence, secrets, the unsaid	· use of humor as a symbolic resource
3. REPERCUSSIONS (in the lives of survivors’ offspring)	· fear of being identified by external indicators	· imaginary resources
		· artistic creation
	· psychical deterritorialization	· appropriation of parental resilience patterns
	· experiences of guilt, victimization and submission	
	· presentification of parental trauma	· field visits and search for knowledge of the Holocaust
	· terrifying worldview	· collective bonding and social support
		· universal values and social and political activism

The *mode of parental psychical working over* concerns survivors’ ability or inability to symbolize disruptive experiences. The pattern of transgenerational transmission or resilience may be regarded as an effect of this phenomenon. When symbolic resources for symbolic working over fail or are lacking, trauma and its transgenerational transmission may lead to several psychopathologic disorders and somatization. The phenomenon of *secondary traumatization* have been described in clinical observations and empirical research, showing that “the consequences of traumatic events are not limited to the persons immediately exposed to the event, and they often affect significant others in their environment such as family, friends, and caregivers. Such effects include difficulties, intrusive imagery, heightened sense of vulnerability, difficulty trusting others, and emotional numbing”. [46].

Otherwise, survivors find diverse means of working over their disruptive experiences, whether by means of personal narratives, documentary records, cultural rituals and expressions, tapping into collective memory or defending universal values. These different modes of appropriation and historicization of experiences may enable the transmission of resilience patterns to the second generation. Nevertheless, a recent study demonstrated that “Holocaust survivors can still display posttraumatic stress symptoms almost 70 years after the trauma, whereas no intergenerational transmission of trauma was found among the second generation” [47].

The *mode of parental psychical working over* is associated with the ways in which survivors relay their Holocaust experiences to their offspring (the *parental communication style*). Our current findings are consistent with previous studies, which indicate that the different roles of affect regulation, narrative cohesion, and symbolic representation, bears the diverse ways in which the parental traumatic experience is appropriated by the offspring [48]. Resilience is a recurring outcome among offspring of families with more open and affectionate communication styles, which frequently make use of humor as a symbolic resource. Conversely, experience of trauma is most evident in offspring of survivors that do not speak of their traumatic experiences, keep them as a secret or relay them in an indirect, fragmented, catastrophic manner.

Some authors prefer the term “echoes of parental traumatic memory” to describe this kind of phenomenon, underscoring that “we are not dealing with the transmission of trauma itself, but rather with interpersonal themes and child-parent dynamics in which these ‘echoes’ of the trauma may play out in the offspring’s recollected relational experiences” [49].

The manner in which the traumatic message is conveyed or silenced by parents can have distinct *repercussions on psychical working over in their offspring.* On the one hand, this message may lead OHS to associate Holocaust trauma with their parents’ inability to adequately carry out their parental functions, compounding potential feelings of psychical deterritorialization. Other patterns of traumatic experience lived by OHS may be manifested in many ways, such as: fear of being identified by external signs; experiences of guilt, victimization and submission; presentification of parental trauma; and a terrifying worldview.

On the other hand, the repercussions of traumatic messages on the lives of OHS may contribute to the development of resilient patterns, when they are associated with the defense of universal or communal values, social and political activism, a search for collective bonding and social support networks, attempts to gain greater knowledge of the Holocaust, artistic creation and realization processes, and exaltation of the radical singularity of parental history – by means of poetic, epic, romanticized narratives –, turning a “cursed inheritance” of sorts into a memorable legacy.

Approaches for the comprehension of transgenerational transmission of trauma have included psychodynamic, cognitive, familiar, social and biologic theories and models. Although being the topic of much research, only very few studies have considered some cultural aspects, like immigration, and their role in the mental health status of OHS. One of these studies postulates that personality differences among OHS could be attributed to their immigrant status rather than to their parents’ experiences as Holocaust survivors [50]. Facing the challenges of being in a new country, with a different culture, could play an important role not only for the first generation, but also for their offspring, who perform the duty of being a facilitator of communication or even a bulkhead between their parents and the local population.

As any qualitative study, findings cannot be generalized. This kind of research has typically a small scale and an exploratory nature. It would be of interest to amplify its domains to other settings, including not only the descendants of victims of wars, but also victims of natural catastrophes and other disasters. This was done in a recent research that studied the mental health consequences of the 2011 Fukushima natural disaster in grandchildren of people living in Hiroshima and Nagasaki during the drop of the atomic bomb [51]. The description of some specific aspects found in the results can also be explored in further researches, with complementary methodologies, like quantitative approaches.

As in other studies of retrospective narratives, the interviewees may highlight their descriptions of past experiences with the colors of present intentions and feelings, with a high level of motivation to talk about their experience that may not be found representative of general population.

Another limitation of this present study is that it did not specify the results in function of the gender of the offspring. This could be of interest, given that some studies have pointed out gender differences in cases of transgenerational transmission of trauma.

## Conclusions

Using a GT approach based on interviews with offspring of Holocaust survivors, this study led to the emergence of a comprehensive model of transgenerational transmission of trauma. Analysis of this model – which is based on phenomena such as survivors’ ability or inability to symbolically work over traumatic events, styles of communication between the first and second generations, and the various repercussions of parental trauma on the lives of their offspring – led to the conclusion that, just as the traumatic dimensions of a traumatic experience can be conveyed transgenerationally, so can the possibility of overcoming trauma, with the development of resilience mechanisms by survivors’ offspring.

As this was a qualitative study, its findings cannot be generalized to other populations. However, exploration of experiences, meanings and subjective processes enabled construction of a theoretical model, which became a relevant theoretical tool for understanding possible mechanisms of transgenerational transmission of trauma and resilience in the offspring of patients with histories of trauma due to catastrophic events. Further studies on this theme are warranted, including studies employing complementary or quantitative methods, in order to confirm or refute the hypotheses generated herein and, possibly, to generalize findings to larger populations.

## Misc

Luciana Lorens Braga Marcelo Feijó Mello José Paulo Fiks contributed equally to this work.

## Competing interests

The authors declare they have no competing interests.

## Authors contributions

LLB conceived the study, performed the design of the research, conducted the interviews and data analysis, and drafted the manuscript. MFM participated in analysis and interpretation of the data and helped to draft the manuscript. JPF participated in the design of the study, in analysis and interpretation of data and critically revised the manuscript. All authors read and approved the final manuscript.

## Pre-publication history

The pre-publication history for this paper can be accessed here:

http://www.biomedcentral.com/1471-244X/12/134/prepub
